# Towards an Understanding of the Function of the Phytochelatin Synthase of *Schistosoma mansoni*

**DOI:** 10.1371/journal.pntd.0002037

**Published:** 2013-01-31

**Authors:** Coraline Rigouin, Elyse Nylin, Alexis A. Cogswell, Dirk Schaumlöffel, Dirk Dobritzsch, David L. Williams

**Affiliations:** 1 Department of Immunology/Microbiology, Rush University Medical Center, Chicago, Illinois, United States of America; 2 Université de Pau et des Pays de l'Adour/CNRS UMR 5254, Laboratoire de Chimie Analytique Bio-Inorganique et Environnement/IPREM, Hélioparc, Pau, France; 3 Martin-Luther-Universität Halle-Wittenberg, Institut für Biochemie und Biotechnologie, Abteilung Pflanzenbiochemie, Halle, Saale, Germany; McGill University, Canada

## Abstract

Phytochelatin synthase (PCS) is a protease-like enzyme that catalyzes the production of metal chelating peptides, the phytochelatins, from glutathione (GSH). In plants, algae, and fungi phytochelatin production is important for metal tolerance and detoxification. PCS proteins also function in xenobiotic metabolism by processing GSH *S*-conjugates. The aim of the present study is to elucidate the role of PCS in the parasitic worm *Schistosoma mansoni*. Recombinant *S. mansoni* PCS proteins expressed in bacteria could both synthesize phytochelatins and hydrolyze various GSH *S*-conjugates. We found that both the N-truncated protein and the N- and C-terminal truncated form of the enzyme (corresponding to only the catalytic domain) work through a thiol-dependant and, notably, metal-independent mechanism for both transpeptidase (phytochelatin synthesis) and peptidase (hydrolysis of GSH *S*-conjugates) activities. PCS transcript abundance was increased by metals and xenobiotics in cultured adult worms. In addition, these treatments were found to increase transcript abundance of other enzymes involved in GSH metabolism. Highest levels of PCS transcripts were identified in the esophageal gland of adult worms. Taken together, these results suggest that *S. mansoni* PCS participates in both metal homoeostasis and xenobiotic metabolism rather than metal detoxification as previously suggested and that the enzyme may be part of a global stress response in the worm. Because humans do not have PCS, this enzyme is of particular interest as a drug target for schistosomiasis.

## Author Summary

Schistosomiasis is a chronic, debilitating disease that affects hundreds of millions of people. The treatment of schistosomiasis relies solely on monotherapy with praziquantel and there is concern that drug-resistant parasites will evolve. Therefore, it is imperative to identify new drugs for schistosomiasis treatment. In this study our goal was to characterize the function of the phytochelatin synthase of *Schistosoma mansoni*, previously suggested as a candidate for drug targeting to control schistosomiasis. Phytochelatin synthase catalyzes the production of metal chelating peptides, the phytochelatins, from glutathione (GSH). In plants, algae, and fungi phytochelatin production is important for metal tolerance and detoxification. PCS proteins also function in the elimination of xenobiotics by processing GSH *S*-conjugates. We found that SmPCS expressed in bacteria could both synthesize phytochelatins and hydrolyze various GSH *S*-conjugates. We found the enzyme works through a thiol-dependant and, notably, metal-independent mechanism for both transpeptidase (phytochelatin synthesis) and peptidase (hydrolysis of GSH *S*-conjugates) activities. The expression of the PCS gene in adult schistosome worms was increased by exposure to a number of metals and xenobiotics. In addition, these treatments were found to increase the expression of other enzymes involved in GSH metabolism. Highest levels of PCS transcripts were localized in the esophageal gland of adult worms. Taken together, these results suggest that *S. mansoni* PCS participates in both metal homoeostasis and xenobiotic metabolism rather than metal detoxification as previously suggested and that it may be part of a global stress response in the worm.

## Introduction

Phytochelatin synthase (PCS) proteins are γ-glutamylcysteine dipetidyltranspeptidases (EC 2.3.2.15) known for their ability to synthesize phytochelatins, which have the general structure (γ-Glu-Cys)_n_-Gly (n≥2, where PC_2_ is a polymer with n = 2; PC_3_, n = 3; etc.), from glutathione (γ-Glu-Cys-Gly; GSH). They have been widely studied in plants, yeasts, algae, and fungi [1]–[6]. More recently they have been identified in bacteria [7], [8] and animals, especially in worms [9]–[12]. Surprisingly, many animals, but not vertebrates, have PCS genes.

A number of studies implicate the activity of phytochelatins in metal detoxification. Phytochelatins are immediately produced after exposure to a range of metal ions, the metal that seems unequivocally involved is cadmium, and their production transforms the metal-sensitive organism to a metal tolerant one [4]. Organisms deficient in PCS display hypersensitivity to cadmium or become unable to tolerate cadmium toxicity [4], [12]. Essential heavy metals such as copper and zinc are required cofactors in redox reactions, ligand interactions and a number of other reactions. However, non essential metals, such as arsenic, cadmium, lead, and mercury are highly reactive and can be toxic through the displacement of endogenous metal cofactors from their binding sites in proteins and in the formation of reactive oxygen species through the Fenton reaction or by the inhibition of enzymes involved in reducing oxidative stress [13]. To control the cellular uptake and to respond to the accumulation of metals, organisms produce metal-binding ligands such as metallothioneins and phytochelatins [2].

Phytochelatin synthases belong to the papain superfamily of cysteine proteases with a mechanism of deglycination of GSH involving a catalytic triad Cys-His-Asp [14]. Most eukaryotic PCS proteins are composed of two domains: i) the N-terminal domain, the catalytic domain for which high sequence homology is found among organisms and ii) the C-terminal domain that has been suggested to function in metal regulation of activity [15]. Most prokaryotic PCS proteins are constituted only of the N-terminal domain. Phytochelatin synthesis involves two distinct reaction steps: the first step involves the cleavage of the glycine from a GSH molecule and generates a γ-Glu-Cys-modified enzyme. In the second step, the γ-Glu-Cys unit is transferred to an acceptor molecule that is either GSH or an oligomeric phytochelatin peptide to generate PC_n+1_ [16], [17].
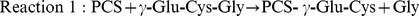


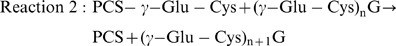



In addition to their role in metal detoxification, PCS proteins are also involved in xenobiotic metabolism and detoxification in plants and fungi [18], [19]. After conjugation of GSH to electrophilic compounds by GSH *S*-transferase (GST), GSH *S*-conjugates are excreted or further metabolized by PCS and/or other enzymes of the phase II xenobiotic degradation pathway.
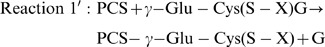


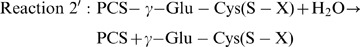



The product of Reaction 2′ can be further processed by γ-glutamyl transpeptidase (γ-GT, EC 2.3.2.2) or cellular proteases prior to excretion as the mercapturic acid derivatives (Cys(S-X)).

Recently, we identified for the first time a PCS in a parasitic organism and a causative agent of schistosomiasis, *Schistosoma mansoni* [11]. Schistosomiasis affects more than 200 million people in more than 70 tropical and sub-tropical countries and causes more than 200,000 deaths annually [20]. Chemotherapy is the major control measure for schistosomiasis and currently only a single drug, praziquantel, is available [21], [22]. Monotherapy for such a widespread and prevalent disease raises serious concerns about the selection of drug resistant parasites [21]. Since schistosomes, but not humans, have PCS its potential as a drug target for the treatment of the disease schistosomiasis has been suggested [11]. Recombinant expression of *S. mansoni* PCS in a yeast system leads an increased cadmium tolerance due to the synthesis of phytochelatins [11]. Because schistosomes, as well as other parasitic flatworms, do not appear to have genes encoding metallothioneins, we have proposed that PCS is the major defense against metal toxicity in this parasite [11], [23]. In addition, because there is a lack of evidence regarding the involvement of phase I detoxification enzymes (e.g. cytochrome P450) in xenobiotic metabolism of trematodes, it is thought that the hydrolytic phase II pathway involving GST conjugation activity to eliminate xenobiotics is mainly used [24], [25]. In this regard, enzymes acting downstream of GST, such as PCS and γ-GT would likely participate in xenobiotic metabolism [26]. However, such activities have yet to be described in the worm to support this hypothesis.

In this paper we have further investigated the molecular mechanisms and the possible functions of PCS in *S. mansoni* (SmPCS). *In vitro* experiments using recombinant SmPCS proteins showed the bifunctionality of the enzyme: SmPCS can synthesize phytochelatins through a thiol-dependant and metal-independent mechanism and can act as a peptidase on GSH *S*-conjugates. To the best of our knowledge, this is the first time that the possible participation of PCS in xenobiotic detoxification in an animal is described. We showed that PCS expression in the parasite is affected by various compounds and that the enzyme may be part of a global stress response. Interestingly, we found PCS transcripts in the esophageal gland suggesting that the action of the enzyme may be for protection against compounds ingested by the worms. Our findings indicate that *S. mansoni* PCS may participate in metal homoeostasis and xenobiotic metabolism rather than metal detoxification.

## Methods

### Ethics statement

This study was approved by the Institutional Animal Care and Use Committee at Rush University Medical Center (IACUC number 11-064; DHHS animal welfare assurance number A3120-01). Rush University Medical Center's Comparative Research Center (CRC) is operated in accordance with the Animal Welfare Act (Public Law (P.L.) 89–544) as amended by P.L.91–579 (1970); P.L.94–279 (1976); P.L. 99–198 (1985); and P.L 101–624 (1990), the Public Health Service's Policy on Humane Care and Use of Laboratory Animals (revised,2002), the Guide for the Care and Use of Laboratory Animals (revised, 2011) and the U.S. Government Principles for the Utilization and Care of Vertebrate Animals Used in Testing, Research and Training. The CRC is registered with the Animal and Plant Health Inspection Service (APHIS) arm of the United States Department of Agriculture (USDA). The Institution has an Animal Welfare Assurance on file with the National Institutes of Health, Office of Laboratory Animal Welfare (OLAW), A-3120- 01. The facilities are accredited by the Association for Assessment and Accreditation of Laboratory Animal Care International (AAALAC International). The CRC is directed by the Senior Director of the CRC, a Doctor of Veterinary Medicine (D.V.M.) and a Diplomate of the American College of Laboratory Animal Medicine (ACLAM), who reports to the Associate Provost and Vice President for Research, who is also the Institutional Official for Animal Care and Use.

### Cloning, expression and purification of SmPCS

**Figure 1:**
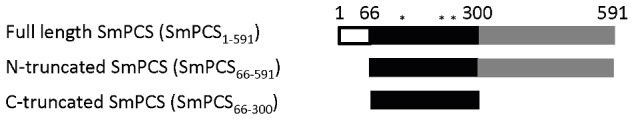
Three different constructions of SmPCS used in the study. The conserved N-terminal PCS region is shown in black, the variable C-terminal region in gray and the predicted mitochondrial peptide signal in white. The asterisks show the position of the three catalytic residues (Cys, His, Asp).

The SmPCS ORF was amplified as previously described [11] and cloned into the pET100 expression vector (Invitrogen). Two other sequences were also cloned into pET100: the N-truncated form corresponding to the amino acids 66 to 590 and the N- and C-truncated form corresponding to the amino acids 66 to 300 (Fig. 1). The resulting constructions (pET100PCS_1–591_; pET100PCS_66–591_ and pET100PCS_66–300_) were used to transform BL21 Star™ (DE3) *Escherichia coli* (Invitrogen).

Recombinant PCS proteins were expressed and purified as described. An overnight culture in Luria broth (LB) containing 50 µg/mL carbenicillin was used to inoculate 1 L LB plus 50 µg/mL carbenicillin culture and the cells were grown at 37°C. After reaching an optical density at 600 nm of 0.5, isopropyl 1-thio-β-D-galactoside was added to 0.1 mM and the culture was incubated overnight at 24°C to express the recombinant protein. After centrifugation at 4500×*g* for 25 min at 4°C, the cells were resuspended in lysis buffer (50 mM potassium phosphate (pH 7.8), 400 mM NaCl, 100 mM KCl, 10% glycerol, 0.5% Triton X-100, 30 mM imidazole, 1 mM dithiothreitol, 1 mM phenylmethylsulfonyl fluoride, 1 mg/mL lysozyme) and disrupted by sonication. After centrifugation at 14000×*g* for 40 min at 4°C, the supernatant was filtered and applied to a 1 mL HisTrap™ HP column (GE Healthcare Life Sciences) first equilibrated with binding buffer (50 mM potassium phosphate (pH 7.8), 400 mM NaCl, 100 mM KCl, 10% glycerol, 0.5% Triton X-100, 30 mM imidazole). The column was washed with 20 mL of binding buffer, contaminant proteins were eluted with 10 mL of elution buffer A (20 mM sodium phosphate (pH 7.5), 500 mM NaCl, 100 mM imidazole, 10 mM β-mercaptoethanol (β-Me)) and the recombinant protein was eluted with 3 mL of elution buffer B (20 mM sodium phosphate (pH 7.5), 500 mM NaCl, 5% glycerol, 500 mM imidazole, 10 mM β-Me). The elution fraction containing the recombinant protein was dialyzed against the same buffer without imidazole. Homogeneity of the purified proteins was confirmed by SDS PAGE. The concentration of proteins was measured according to the method of Bradford [27].

### Enzyme assays

PCS activity was measured according to the method of Oven *et al.* [28]. The reaction mixture (180 µL) contained 200 mM Tris-HCl (pH 8), 10 mM β-Me, 10 mM GSH, 0.1 mM CdCl_2_ and 3 µg of SmPCS. PCS activity for PC_2_ synthesis was measured in the same condition but in a final volume of 60 µL. PCS activity was expressed as the amount of PC_2_ synthesized by 1 mg of protein per minute and was measured in the first 15 min when the synthesis of PC_n_ (with n>2) was negligible. In the conditions without added metals, the chelating agent diethylene triamine pentaacetic acid (DTPA) (6.3 mM) was added to the reaction mix to chelate any metals carried over from expression and purification of the protein. To measure PCS activity with GSH *S*-conjugates, the reaction mixture (60 µL) contained 200 mM Tris-HCl (pH 8), 10 mM β-Me, 1 mM GSH *S*-conjugate, 0.1 mM CdCl_2_ and 3 µg of SmPCS. For the metal sensitivity experiments, various concentrations of CdCl_2_ or ZnCl_2_ were used. All the reactions were incubated at 37°C and were stopped by addition of an equal volume of 0.2 N HCl. After a centrifugation at 12,000×*g* for 5 min, the supernatants were analyzed by LC-MS.

### Synthesis of GSH *S*-conjugates

For the synthesis of GSH *S*-bimane (GS-bimane), the reaction mixture contained 10 mM monobromobimane prepared in 100% acetonitrile, 10 mM GSH, 200 mM HEPPS buffer (pH 8) containing 6.3 mM DTPA. The mixture was incubated at 45°C for 30 min in the dark and the reaction was terminated by the addition of 1 M methane sulfonic acid to achieve a final concentration of 0.1 M. The reaction was then dried in a speed vacuum and the pellet resuspended in 25 mM Tris-HCl pH 8 and filtered to remove the excess of monobromobimane. For the synthesis of GSH *S*-ethacrynic acid (GS-EA), the reaction mixture contained 10 mM GSH, 0.2 mM ethacrynic acid prepared in 70% ethanol and 100 mM potassium phosphate buffer pH 7. The mixture was incubated at 37°C for 30 min and used without further purification.

### LC-MS

Twenty µL of each sample was injected onto a reverse-phase column (Eclipse Plus C18, Agilent) attached to an Agilent 1946A LC-MSD system, an Agilent 1100 HPLC LC system coupled to a single quadrupole LC-mass spectrometer equipped with electrospray ionization (ESI). Thiol compounds were separated by using solvent (A) 0.1% TFA and (B) 100% acetonitrile at a flow rate of 0.5 mL/min. The gradient was from 2% to 20% of solvent (B) over 20 min. Before injecting a new sample, the column was washed (4 min 98% B) and equilibrated (4 min 2% B). The integrated peak area was used to quantify the PC levels after calibration with chemically synthesized PC_2_, PC_3_, and PC_4_ (AnaSpec). PCS activity was expressed as the amount (µmol) of phytochelatins synthesized by 1 mg of enzyme. For the analysis of the hydrolysis reaction of GSH *S*-bimane (or GSH *S*-EA), the gradient used was from 30% to 45% of solvent (B) over 8 min. The integrated peak area of the extracted ion chromatogram was used to quantify γ-Glu-Cys-*S*-bimane levels after calibration with synthetic GSH *S*-bimane. PCS activity was expressed as the amount (µmol) of γ-Glu-Cys-*S*-bimane synthesized by 1 mg of enzyme.

### *Ex vivo* worm culture

Perfusions of adult worms (6–7 wk post infection) from mice were done as previously described [29]. Adult worms collected from mice were incubated in complete DMEM medium (Difco) containing 100 U/mL penicillin, 100 µg/mL streptomycin and 10% heat-inactivated FBS. For the treatments, six pairs of worms per well were incubated in 6-well tissue culture plates at 37°C with 5% CO_2_. Metals (CdCl_2_, FeCl_3_ and ZnCl_2_) were added at a final concentration of 100 µM. Worms were exposed to 30 µM monobromobimane (Sigma), 1 µg/mL praziquantel (Sigma), 10 µM ethacrynic acid (MP Biomedical Inc.), or 100 µM H_2_O_2_. Six hour after addition of the compounds, worms were collected, washed twice in PBS, flash frozen in a dry ice/ethanol bath and stored at −80°C. Parasites without treatment were incubated the same length of time and used as controls.

### cDNA synthesis and qPCR

First strand cDNA was synthesized from adult worm total RNA. RNA was extracted from worms using TRIzol® (Invitrogen) according to the manufacturer's instructions. cDNA was synthesized using the *iScript* cDNA synthesis kit (Bio-Rad) using 1 µg of total RNA. For quantitative PCR experiments, 1 µg of cDNA was used with the SsoFast™ EvaGreen® Supermix in an ABI PRISM 7900 sequence detection system (Applied Biosystems). qPCR primers were designed for SmPCS SmGST26, SmGST28, SmγGT and SmγCGL and for the housekeeping gene β-tubulin using PrimerQuest (IDT) (Table 1). Fold differences were calculated using the 2^−ΔΔCt^ method [30].

**Table 1:** List of the primers used for RT-PCR analysis.

Gene	Accession number	Primer	Sequence
γ-CGS	Smp_013860	γ-GCS F	5′ACGATCAGCTAGCAACAATGTGCC3′
		γ-GCS R	5′AACGGCAGTCCCAATCCAGTAAGT3′
β-tubulin	Smp_078040	β-tubulin F	5′TGCCTCGTGCTATTCTGGTTGACT3′
		β-tubulin R	5′AATTATCTGGCCGGAACAACTGCC3′
GST26	Smp_102070	GST26 F	5′AGGCCTTGTACAACCAACTCGTCT3′
		GST26 R	5′ACATCGCCGTCATTGCGATCATAC3′
GST28	Smp_054160	GST28 F	5′ATGACTCTTGTGGCAGCTGGTGTA3′
		GST28 R	5′ATCGTCCGCCTGGAATAGTTGGTT3′
γ-GT	Smp_089100	γ-GT F	5′GGTGCTGTTGCAGTTGACGATGAT3′
		γ-GT R	5′CCATCGCATCAACAGCATTCCCTT3′
PCS	Smp_072740	PCS F	5′TTGACGTGGTTGACTTCTGACGGT3′
		PCS R	5′GTGAAAGGGCAATCATCAGTGGGT3′
TGR	Smp_048430	TGR F	5′ACCTTTAGAATGGACCGTCGCTCA3′
		TGR R	5′ACCCAGTACACGCATGTTGTCAGA3′

Smp = Schistosoma mansoni GeneDB accession number (http://www.genedb.org/Homepage/Smansoni).

### Whole mount *in situ* hybridization (WISH)

Riboprobes were synthesized according to previously published methods [31]. Briefly, probes were synthesized from restriction enzyme digested DNA according to the orientation of the transcript in pCRII, using the Riboprobe synthesis kit (Promega) with SP6 or T7 polymerases and the digoxigenin (DIG) RNA labeling kit (Roche). WISH was also done according to previously published methods [31].

### HPLC (Ellman) and UPLC-MS/MS analysis of worm homogenates for phytochelatins

For preparation of the worm extracts the lyophilized worm homogenates were extracted 1∶4 with 0.1 N HCl, vortexed, centrifuged (9,500×*g*, 12 min, 4°C), filtered and immediately analyzed by HPLC. Prior to UPLC-MS 500 mM tris(2-carboxyethyl)phosphine were added to the worm extracts in the ratio 1∶29 in order to reduce potentially present phytochelatins.

HPLC (Ellman) was conducted on a RP18 column (Bischoff, Leonberg, ProntoSil C18 AQ, 5 µm 120 Å; 250×4.6 mm) via a Merck-Hitachi La Chrom HPLC system (Darmstadt, interface d-700, pump L-7110/L-7100, autosampler L-7200, UV/Vis detector L-7420). All solvents used were helium degassed. The mobile phase A consisted of trifluoroacetic acid in water (pH 2) and a mobile phase B of acetonitrile with a flow rate of 1 mL min^−1^. The injection volume of the samples was 70 µL. A linear gradient from 2 to 20% B during 20 min followed by isocratic elution at 20% B during 5 min was applied. For thiol-specific detection, postcolumn derivatization was carried out by adding via a T-piece 0.4 mL min^−1^ 300 µM Ellman's reagent (DTNB) in 50 mM KH_2_PO_4_, pH 8.0, 1-mL reaction loop). Detection was performed at λ = 410 nm.

For UPLC-MS/MS analysis a nanoUPLC – MS system from Waters (Eschborn, Germany) was operated in “single pump trapping” mode. Therefore, analytes were preconcentrated on a C18 trapping column during 1 min with an eluent flow rate of 5 µL min^−1^ under isocratic conditions (1% acetonitrile and 0.1% trifluoroacetic acid in water). Via a nano-Tee-valve the eluent was directed into the waste. Afterwards the flow rate was decreased to 0.3 µL min^−1^ and nano-Tee-valve was switched directing the eluent on the analytical column. A linear gradient from 3 to 50% acetonitrile during 30 min was used. Analytes in the UPLC eluent were ionized by electrospray at 2.5 kV and introduced into the mass spectrometer. Ions were scanned in the range *m/z* 100–1500 for acquiring MS spectra. Selected masses were fragmented by collision induced dissociation with energies between 10 and 60 V. The acquired data was analyzed with the Waters MassLynx 4.1 update 3 software.

## Results

### Recombinant SmPCS synthesizes phytochelatins

**Figure 2:**
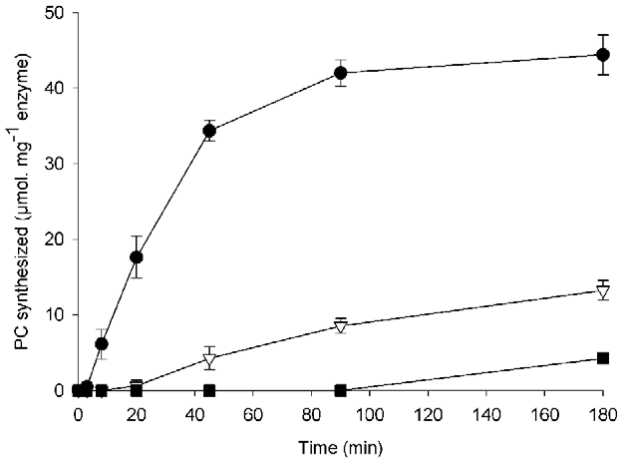
Synthesis of PC_2_, PC_3_ and PC_4_ by SmPCS_66–591_. The incubation mixture contained 200 mM Tris-HCl (pH 8), 10 mM β-Me, 10 mM GSH, 0.1 mM CdCl_2_ and 4 µg of purified SmPCS. PC_2_ (black circle), PC_3_ (open triangle) and PC_4_ (black square) are expressed in µmol per mg enzyme, as quantified by LC-MS. Mean value of three separate assays (± SE) are given.

Three different forms of the *S. mansoni* PCS protein were produced in *E. coli*: the full length protein (PCS_1–591_), the N-truncated protein (PCS_66–591_) and the C-truncated protein (PCS_66–300_) (Figure 1). All three purified, six-His-tagged recombinant proteins were successfully expressed and purified to homogeneity at 1 mg/L culture and formed homodimers as determined by size exclusion chromatography (data not shown). Recombinant PCS_1–591_, corresponding to the unprocessed, mitochondrial form of the protein [11], was enzymatically inactive for the production of phytochelatins. This suggests that the presence of the predicted mitochondrial signal peptide in the protein when expressed in *E. coli* may prevent correct protein folding. Recombinant SmPCS_66–591_, corresponding to the cytoplasmic form of the protein, was found to be active in phytochelatin synthesis (Figure 2). In the first 15 min of the reaction, SmPCS_66–591_ synthesizes PC_2_, PC_3_ appears after 20 min incubation, and PC_4_ after 90 min when PC_2_ synthesis reaches a plateau. PC_5_, PC_6_, and PC_7_ were detected after 180 min indicating that SmPCS can synthesize high molecular weight species of phytochelatins (data not shown).

During the time course analysis, no accumulation of γ-Glu-Cys was detected. This suggests that once cleaved from GSH, γ-Glu-Cys is immediately consumed for the synthesis of phytochelatins. To compare the catalytic mechanism of SmPCS to PCS proteins from other organisms, the reaction was also carried out with 1 mM synthetic PC_2_ as the sole substrate. In this case, we were able to detect PC_3_, γ-Glu-Cys and GSH after 60 min incubation (data not shown). Therefore, as previously described for other PCS proteins [8], SmPCS synthesizes PC_3_ through the cleavage of PC_2_ into γ-Glu-Cys and GSH followed by the conjugation of γ-Glu-Cys to another PC_2_ molecule.

To define the catalytic domain of SmPCS we used a C-truncated form of the protein, SmPCS_66–300_. Phytochelatin synthesis (PC_2_, PC_3_, and PC_4_) by SmPCS_66–300_ was found to be the same as by the full length protein (PCS activity for PC_2_ synthesis by SmPCS_66–591_ = 0.88±0.14 µmol min^−1^ mg^−1^ and by SmPCS_66–300_ = 1.2±0.14 µmol min^−1^ mg^−1^). Therefore, the N-terminal domain is sufficient for efficient PC synthesis and the C-terminal domain does not enhance the catalytic activity of SmPCS *in vitro*.

### SmPCS is active without a metal cofactor but requires a reducing agent

**Figure 3:**
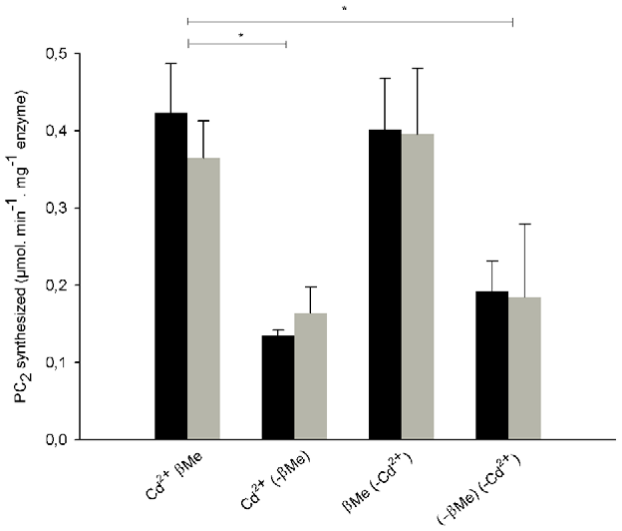
PC_66–591_ (light grey) and PC_66–300_ (dark grey) activities for PC_2_ synthesis. The incubation mixture contained 200 mM Tris-HCl (pH 8), 10 mM GSH, 3 µg of purified SmPCS and either 0.1 mM CdCl_2_ or the chelating agent diethylene triamine pentaacetic acid (6.3 mM). β-mercaptoethanol (β-Me) was added to 10 mM as indicated. PC_2_ is expressed in µmol per mg enzyme per min, as quantified by LC-MS. Mean value of three separate assays (± SE) are given. *, p<0.05 by Student's t test.

To investigate the influence of metals on SmPCS activation, we followed PC_2_ synthesis with either SmPCS_66–591_ or SmPCS_66–300_ with cadmium added to the reaction for the condition with metal or with DTPA added to chelate any residual metals for the condition without metal. Surprisingly, we found that that enzymatic activity was the same in the absence of metals (+DTPA) as in the presence of 100 µM of cadmium (−DTPA) and PC_2_ synthesis is inhibited in the absence of a reducing agent (Fig. 3). To investigate the importance of the reducing agent for the enzyme activity, we used *S*-methyl-GSH as sole substrate for the enzyme since the GSH through its thiol group can play the role of reducing agent. We found that both forms of enzyme were capable of synthesizing *S*-methyl-phytochelatins from *S*-methyl-GSH only in the presence of β-Me or the non-thiol reducing agent *tris*(2-carboxyethyl)phosphine) and independently of the presence of cadmium (data not shown). Therefore, the presence of a reducing agent, but not the presence of metal, is necessary for SmPCS activity.

To further investigate the effects of metals on PCS activity, we followed PC_2_ synthesis by SmPCS_66–591_ and SmPCS_66–300_ with increasing concentrations of cadmium and zinc (Table 2). For the full length protein, we found that PC_2_ synthesis decreased at higher concentrations of cadmium (69±7% of activity with 1 mM CdCl_2_ in the reaction mixture). With addition of zinc, full activity was obtained with only 20 µM of metal and above this concentration the activity decreased significantly. Activity for the C-truncated form of the enzyme did not change significantly with increasing concentration of metal. These results suggest that in addition to the lack of a requirement for a metal cofactor, metals can inhibit PCS activity at high concentrations.

**Table 2:** Relative activity of SmPCS_66–591_ and SmPCS_66–300_ with different metals.

Metal	Concentration (mM)	Relative activity (%)	
		PCS_66–590_	PCS_66–300_
CdCl_2_	0.1	100±23	94±23
	0.5	87±7	125±14
	1	69±7*	87±43
ZnCl_2_	0.02	131±44	90±44
	0.1	23±3*	60±33

Values are given as the percentage of activity (PC_2_ synthesized µmol min^−1^ mg^−1^ enzyme) of respective enzyme in the absence of added metal in the presence of DTPA (6.3 mM). Mean value of three separate assays (± SE) are given (N = 3). The incubation mixtures contained 200 mM Tris-HCl (pH 8), 10 mM β-Me, 10 mM GSH, 3 µg of enzyme and the metal ion.

* , p<0.05 by Student's t test.

### Recombinant SmPCS hydrolyses GSH *S*-conjugates

**Figure 4:**
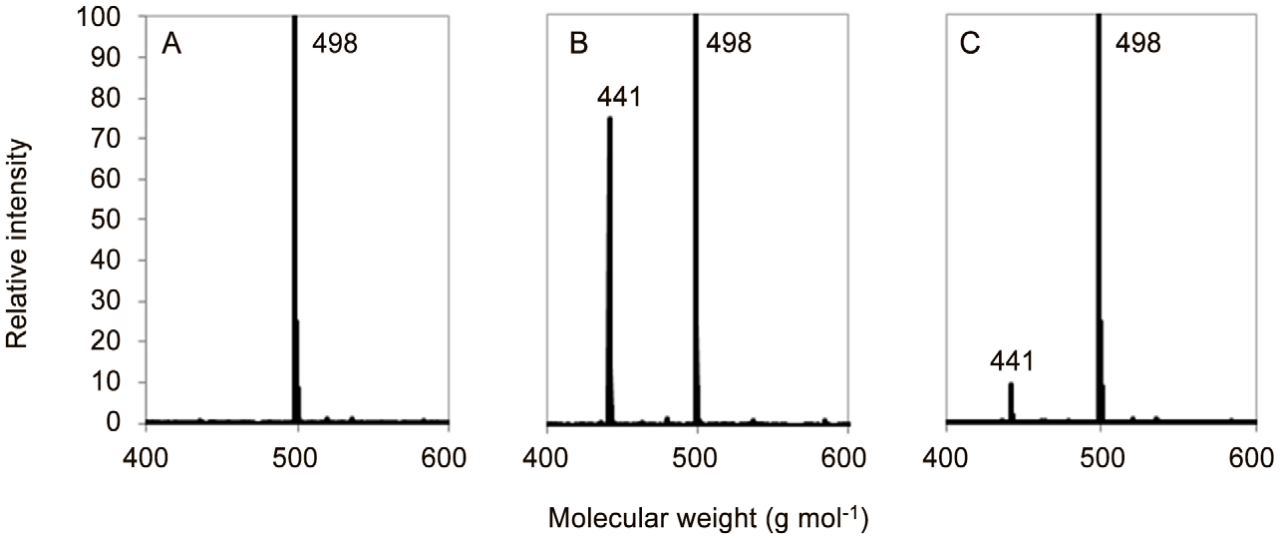
Peptidase activity of PC_66–591_ and PC_66–300_. LC-ESI-MS product-ion mass spectra of the reaction mixtures incubated at 37°C for 30 min (A) 1 mM GS-bimane, 10 mM β-Me, 0.1 mM CdCl_2_, 200 mM Tris-HCl (pH8) (GS-bimane *m/z* 498) and (B) 1 mM GS-bimane, 10 mM β-Me, 0.1 mM CdCl_2_, 200 mM Tris-HCl pH8 and 5 µg of recombinant SmPCS_66–300_ (γ-Glu-Cys-*S*-bimane *m/z* 441) and (C) 1 mM GS-bimane, 10 mM β-Me, 0.1 mM CdCl_2_, 200 mM Tris-HCl pH8 and 5 µg of recombinant SmPCS_66–591_.

We next investigated whether the enzyme was able to act as a peptidase on GSH *S*-conjugates. Monobromobimane (bimane), a compound that labels thiols, was used. This compounds is known to conjugate to GSH both enzymatically (by GST) and non-enzymatically, *in vitro* as well as *in vivo*. The GSH *S*-conjugates, GS-bimane, was synthesized and tested as substrate for SmPCS_66–300_ and SmPCS_66–591_. The reactions were analyzed by LC-MS for the appearance of the product γ-Glu-Cys-S-bimane. We found that both SmPCS_66–591_ and SmPCS_66–300_ cleaved the glycine from the GSH conjugate to give the corresponding γ-Glu-Cys-*S*-conjugate. Indeed, the mass spectra of the control reactions (substrate alone) exhibited the [M+H] ion of GS-bimane (*m/z* 498) (Fig. 4A). In the presence of SmPCS_66–591_ and SmPCS_66–300_, the mass spectra displayed an additional peak at *m/z* 441 corresponding to γ-Glu-Cys-*S*-bimane (Fig. 4B and 4C, respectively). Using extracted ions chromatogram we could measure the respective amount of GS-bimane and γ-Glu-Cys-*S*-bimane that are co-eluted by LC-MS. We found that SmPCS_66–300_ and SmPCS_66–591_ activity for γ-Glu-Cys-*S*-bimane synthesis was 109.6±5.3 and 11.7±4.2 µmol min^−1^ mg^−1^ respectively. This suggests that the N-terminal domain of the enzyme alone is more efficient in the catalytic cleavage of GS-conjugates than the full length enzyme. Production of the hydrolyzed products was detected in the presence and in the absence of added metals but no cleavage occurred in the absence of the reducing agent β-Me (data not shown). Similar results were obtained with GS-EA (data not shown).

### SmPCS is upregulated at a transcriptional level when worms are exposed to various stressors

**Figure 5:**
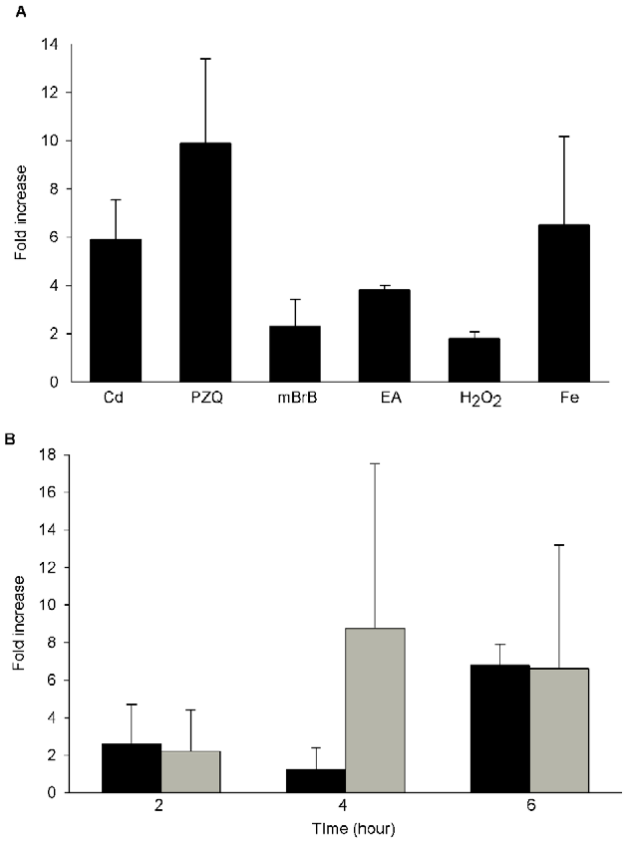
Real-time RT-PCR analysis of PCS in response to various stresses. **A**. Adult worms were exposed to different stressors (Fe: 100 µM FeCl_3_; Cd: 100 µM CdCl_2_; PZQ: 1 µg/mL praziquantel; mBrB: 30 µM monobromobimane; EA: 10 µM ethacrynic acid; H_2_O_2_: 100 µM hydrogen peroxide) for 6 h. SmPCS mRNA abundance was determined by real-time RT-PCR. **B**. Real-time RT-PCR analysis of PCS in response to metal with time. Adult worms were exposed to 100 µM FeCl_3_ (dark grey) or 100 µM CdCl_2_ (light grey) during 2, 4 and 6 h. SmPCS mRNA abundance was determined by real-time RT-PCR.

Phytochelatin synthase activity is closely related to metal abundance through the synthesis of the metal-chelating PC peptides and to xenobiotic metabolism through its peptidase activity on GS-conjugates. To understand the function of the enzyme in the parasite, we investigated the transcriptional regulation of the enzyme by analyzing the level of SmPCS mRNA transcripts in adult worms exposed to metals and xenobiotics. Worms were cultured in the presence of stressors for 6 h and the abundance of SmPCS mRNA was evaluated by quantitative real-time reverse transcription-PCR (qPCR). The level of mRNA of SmPCS increased 5.9±1.6 fold in the presence of cadmium and 6.4±3.6 fold with iron compared to the control (Fig. 5a) indicating that worms respond to metal exposure by synthesizing PCS. The time course of induction was similar with both metals (Fig. 5b). Some xenobiotics are known to be conjugated to GSH by GSTs and thus potentially substrates of PCS. Therefore, monobromobimane and ethacrynic acid (EA) acid were tested for their ability to alter SmPCS expression. We found that both compounds caused an increase in mRNA levels of SmPCS after 6 h, 2.3±1.1 and 3.8±0.2, respectively, suggesting that adult worms synthesize PCS also in response to xenobiotics. The drug used clinically to treat schistosomiasis, praziquantel, was also tested since it is known to increase the expression of many genes in the worms [32]. Interestingly, although praziquantel is not believed to be processed by GST conjugation and may trigger different pathway of elimination, it was found to increase SmPCS mRNA abundance (9.9±3.5). To test whether oxidative stress would have an effect on PCS mRNA abundance, worms were treated with H_2_O_2_ and PCS transcripts were found to be increased 1.8±0.2 fold compared to the control. Taken together, our data demonstrate that PCS transcripts are upregulated by obvious inducers as well as less evident stressors.

### Various stressors increase the transcription of genes involved in the glutathione metabolism pathway

**Figure 6:**
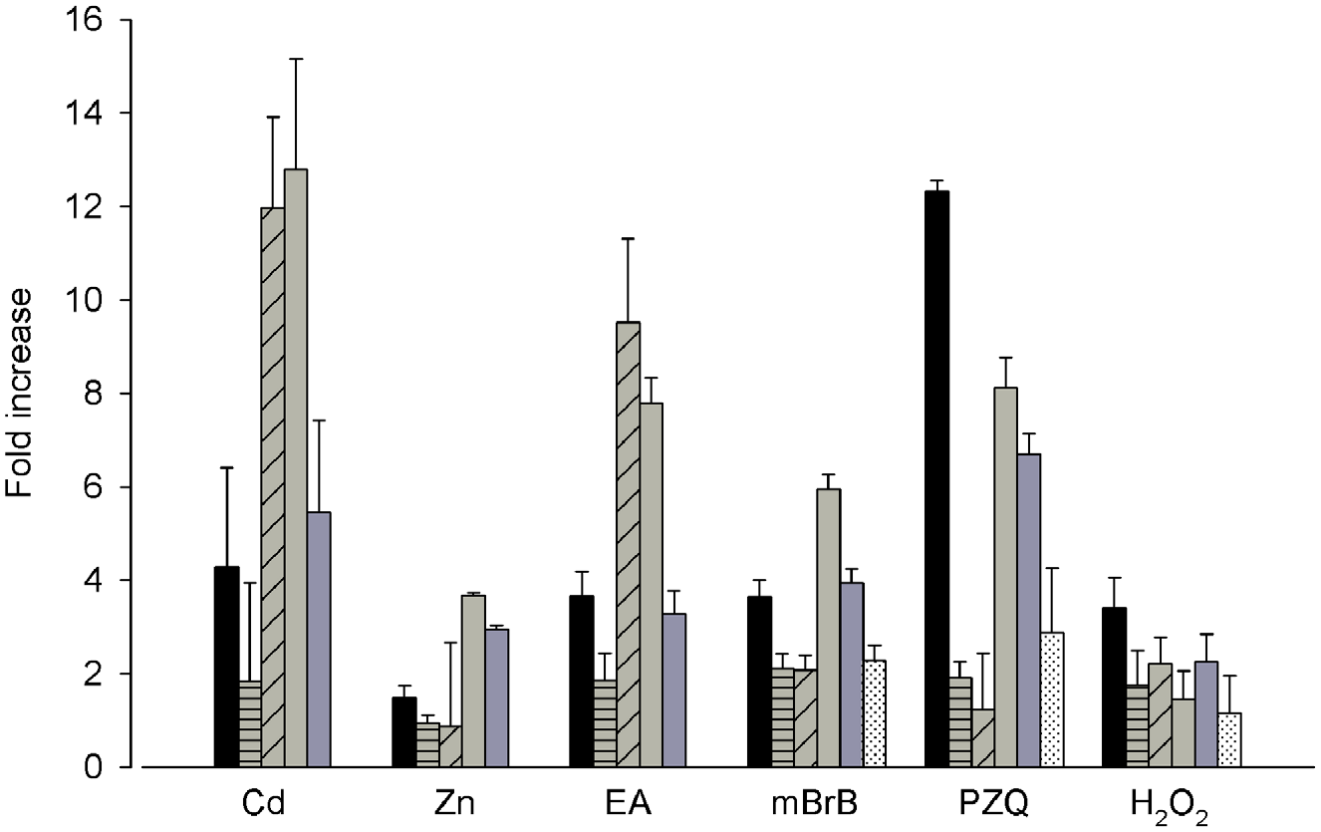
Real-time RT-PCR analysis of glutathione metabolic enzymes in response to various stresses. Adult worms were exposed to Cd: 100 µM CdCl_2_; Zn: 100 µM ZnCl_2_; EA: 10 µM Ethacrynic acid; mBrB: 30 µM monobromobimane; PZQ: 1 µg/mL praziquantel; H_2_O_2_: 100 µM hydrogen peroxide for 6 h. Relative mRNA abundance of PCS (black bar); GST26 (light grey horizontal bar), GST28 (light grey hatched bar), γGT (light grey plain bar); γGCS (dark grey bar); TGR (white bar with black dots) were determined by real-time RT-PCR.

We next investigated if changes in PCS expression were coordinated with the expression of other genes involved in GSH metabolism. Our hypothesis was that the worms produce PCS not specifically to detoxify metals but rather as part as a systemic stress response. Changes in the mRNA abundance of two GSTs, GST26 and GST28, γ-GT, γ-glutamylcysteine synthetase (γ-GCS), the rate-limiting enzyme in GSH synthesis, and thioredoxin glutathione reductase (TGR) in worms treated for 6 h with different stressors were monitored by qPCR (Fig. 6). Most of the stressors that were found to increase PCS mRNA abundance also increased γ-GT and γ-GCL mRNA levels. γ-GCS transcript levels increased in response to all treatments indicating that GSH synthesis is required to cope with metal and xenobiotic exposures and oxidative stress. Although H_2_O_2_ did not enhance TGR mRNA level as might be expected since it is an antioxidant enzyme, it did increase PCS and γ-GCS mRNA levels suggesting that GSH synthesis may be the first cellular response to oxidative stress. Interestingly, praziquantel was found to increase the level of all transcripts tested, suggesting that as previously mentioned, schistosomes exposed to praziquantel may undergo a transcriptomic response similar to that observed during oxidative stress [32]. In addition, some of the treatments (cadmium, EA, monobromobimane) also triggered GST transcription and in particular, GST28. This suggests that the cell copes with these stressors by triggering GSH metabolizing pathways. Overall, this set of data support the hypothesis that SmPCS is part of the protective response of the organism utilizing GSH.

### Phytochelatins in worms

Cadmium-treated and control worm samples were investigated for the presence of phytochelatins. Classic thiol detection by HPLC and Ellman post-column derivatization as well as modern phytochelatin analysis by UPLC-MS was applied. The latter method offers highly specific and ultrasensitive phytochelatin detection by their exact masses in MS and fragment ions in MS/MS. Detection limits down to 5 and 17 nM for PC_2_ and PC_3_, respectively, were achieved in a previous study [33]. In the present study both methods were optimized with a PC_2_ standard. HPLC (Ellman) confirmed the presence of GSH in worms, but no phytochelatins could be detected. UPLC-MS was optimized for phytochelatin detection, but not for GSH, showed no signal for phytochelatins in the investigated worm samples although with the same system traces of PC_2_ could be recently detected in chromium stressed algae (unpublished data). In conclusion, phytochelatins could not be detected down to the nM level in metal treated worms.

### PCS transcripts localize in gut and esophageal gland of both male and female adult worms

**Figure 7:**
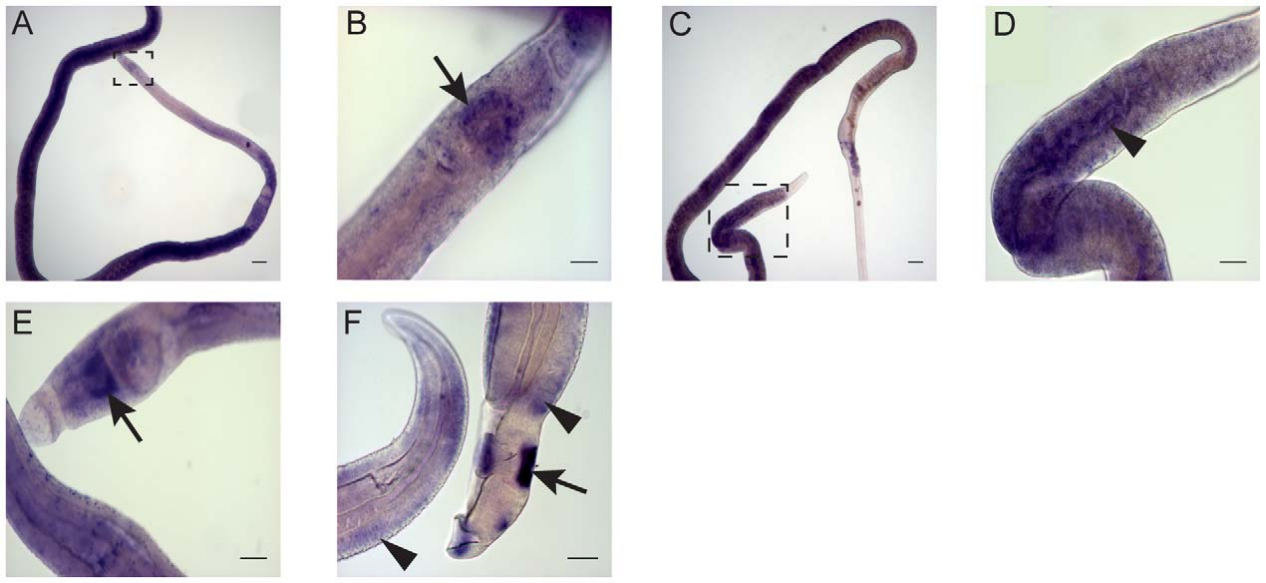
Tissue localization of phytochelatin synthase expression by WISH. Boxed regions in A and C are shown at higher magnifications in B and D. Gut (arrowheads) and esophageal glands (arrows) localization are indicated in female (A–D) and male (E–F) parasites. All images were generated using an AxioStar Plus microscope and analyzed with ImageJ software. Scale bars are 100 µm in A, C, E, and F, 25 µm in B, and 50 µm in D.

Whole mount *in situ* hybridization (WISH) was used to localize PCS transcripts in adult worms. Digoxigenin labeled probes designed to the C-terminal sequence of PCS were generated and used for WISH. PCS transcripts localize to the gut epithelium and esophageal gland in adult male and female worms (Fig. 7).

## Discussion

In this study, we set out to understand the catalytic processes of SmPCS and its function in the parasite. Analysis of recombinant SmPCS allowed us to highlight the characteristics of this unusual enzyme. The recombinant full length protein did not have enzymatic activity. It is likely that the mitochondrial peptide prevents the correct folding of the protein in *E. coli*. The N-truncated protein, SmPCS_66–590_, corresponding to the cytoplasmic form of the enzyme in the parasite, lacks the amino acids corresponding to the predicted mitochondrial targeting peptide [11]. We showed that SmPCS is capable of synthesizing phytochelatins as previously suggested [11]. We found that the enzyme also functions as a peptidase using GSH *S*-conjugates as substrates. Although those two activities have been described for PCS enzymes from other organisms, significant differences in the molecular mechanisms between SmPCS and other PCS proteins were observed.

The molecular mechanism of PCS catalysis is not clear and there are contradictions between studies regarding metal activation and the role of the C-terminal domain. Vatamniuk *et al.* used as a model the recombinant flag-tagged PCS from *Arabidopsis thaliana* (AtPCS-flag) and described that during the first step acyl-enzyme intermediate formation occurs at two distinct sites [16]. They proposed that the first acylation occurs at the N-terminal domain of the enzyme and does not require metal and that the second acylation occurs at a site located in the C-terminal domain and is metal dependant. Romanyuk *et al.* suggested that the C-terminal domain is required for second site acylation and metal sensing and augments the catalytic process [34]. Although it is clear that the N-terminal domain is sufficient for the catalysis [8], [15], [34], the position of the second acylation site is not well established.

The second point of contradiction is the involvement of metals in PCS activity. It has been suggested that although the enzyme requires metal to synthesize phytochelatins, it is not a metal in a free state that interacts directly with the enzyme leading to its activation but the formation of a metal complex with GSH (e.g., GS-Cd_2_) that functions as the substrate [17]. However, other studies also using AtPCS as a model have demonstrated a direct interaction of the enzyme with the metal [28], [35].

Although previous studies investigating PCS activity showed that PCS proteins with both N- and C-terminal domains have an absolute requirement for metals for GSH-dependent phytochelatin synthesis activity [4], PCS from cyanobacteria, which has only the N-terminal domain, has been shown to function without added metals [14]. Our results indicate that neither the SmPCS protein with both the catalytic and C-terminal domains (SmPCS66-591) nor the N-terminal-only protein (SmPCS66-300) require metal for its activity. In our experiments, chelation of metals by DTPA did not affect SmPCS activity and thus it is likely that SmPCS activity is independent of metal ions. However, the fact that both SmPCS and AtPCS, can catalyze the synthesis of *S*-methyl PC from *S*-methyl GSH independently of metal suggests that no direct interaction of the metal with the enzyme is required [17], [34]. In the case of AtPCS, blocked GSH (S-methyl GSH or GS-metal complexes) appear to serve as substrates, while this does not appear to hold for SmPCS. It is noteworthy that an increase in metal concentration was found to inactivate SmPCS_66–591_ but not SmPCS_66–300_, suggesting that metals can interact with the C-terminal domain resulting in enzyme inactivation. It has been suggested that AtPCS possesses a binding site where metal binds resulting enzyme inhibition [35], but whether or not this is true for SmPCS remains to be determined.

Our data show that the C-terminal domain of SmPCS does not participate in metal sensing as previously suggested for other PCS enzymes [15] or provide a second acylation site since SmPCS_66–300_ retains the same phytochelatin synthetic activity as SmPCS_66–590_. In our previous study, it was found that a truncated form of the protein (deletion of the 100 C-terminal amino acids) did not provide cadmium tolerance when expressed in yeast suggesting that no PC synthesis occurred [11]. In this study, the deletion was made so that the C-truncated form would match with the sequence of Nostoc PCS. It is possible that the 100 amino acid C-terminal truncated SmPCS protein studied previously does not fold correctly or is unstable when expressed in yeast. Another possibility could be that the C-truncated form does not behave similarly *in vitro* and *in vivo* as is the case for AtPCS [8]. A cysteine residue (Cys109 in AtPCS) is conserved in most of the plant, algal, and animal cadmium-dependant PCS proteins [11]. This particular residue is not found in SmPCS (Lys) nor in Nostoc PCS (Lys), PCS proteins that appear to have metal-independent activity. This particular cysteine may be involved in metal sensing and could be a cadmium-dependant acylation site. Mutant PCS proteins with a deletion of this cysteine still have phytochelatin synthesis activity [16], but no investigations concerning the metal-dependent activity of these mutants have been done. The role of the C-terminal domain in the synthesis of phytochelatins by SmPCS is not clear, but it appears to be dispensable.

Although the role of reducing agents in PCS activity has not been extensively documented, previous studies have shown that AtPCS has lower activity in the absence of a reducing agent while Nostoc PCS activity is independent of added thiols [8], [28]. We found that a reducing agent was required for SmPCS activity. AtPCS and SmPCS display, respectively, seven and eight cysteine residues in their N-terminal domain and eight cysteine residues in their C-terminal domain and require a reducing agent for their activity. Interestingly, Nostoc PCS has only one cysteine (the catalytic residue) and does not require a reducing agent to be active [8]. Therefore, the abundance in cysteine in both domains may be correlated with a requirement for a reducing agent; the enzyme must be in a reduced state to be active. In this regard, it would be interesting to study the sensitivity of the Cys mutant proteins generated by Tsuji *et al.* or Vatamaniuk *et al* toward reducing agents [8], [16]. Our observation that in high concentrations of metals SmPCS is inhibited suggests that the enzyme with reduced thiols may chelate metals and adopt an inactive conformation, perhaps similar to the oxidized form of the protein. Thus, reducing agents for the schistosome enzyme and metals for other PCS proteins act similarly resulting in protein activity; perhaps reducing agents mimic cadmium, which normally binds to the auxiliary cysteines stabilizing protein function.

The active site amino acid triad – Cys-His-Asp – is contained in SmPCS_66–300_, full-length Nostoc PCS, and AtPCS_1–221_, the C-truncated form of AtPCS [14]. SmPCS and Nostoc PCS catalyze PC_2_ synthesis at the similar rates, 1.1 µmol SH mg^−1^ protein min^−1^ [8] and 0.84 µmol SH mg^−1^ protein min^−1^ (Fig. 3), respectively. The full length AtPCS activity is 14 µmol SH mg^−1^ protein min^−1^ but is only 5 µmol SH mg^−1^ protein min^−1^ for the C-truncated form. This suggests that the enzymes that do not require cadmium are less active than those that are cadmium-dependant. Or, as suggested by Vatamaniuk *et al.*, the C-terminal domain of those enzymes may contain the acylation site (cadmium-dependant) that would enhance the activity. The less-conserved Cys residues in the C-terminal domain, often presented in pairs in cadmium-dependant PCS proteins, may have a role as binding sensors for metals [36].

Interestingly, we found that SmPCS can catalyze the cleavage of the glycine residue from GSH *S*-conjugates. This is the first time that the peptidase activity of a PCS from animal has been described. We found that metal and reducing agent requirements for the peptidase activity are similar to that of the transpeptidase activity. The C-truncated form of the protein appeared to be more efficient in the cleavage reaction. This is probably due to a better access of the large substrates that represent GS-conjugates in the binding pocket. The peptidase activity was detected with compounds known to be processed *in vivo* by GST in the process of xenobiotic metabolism. Because in schistosomes the main elimination pathway is thought to be through GST conjugation [37] it is likely that *in vivo* PCS acts in tandem with γ-GT to process GSH *S*-conjugates. GSH conjugation and subsequent peptidase processing are also involved in the metabolism of endogenous compounds such as leukotrienes [38]. Whether SmPCS peptidase activity is involved *in vivo* in xenobiotic detoxification or in the synthesis of endogenous compounds has yet to be determined.

*In vivo*, we have shown that metals such as cadmium and iron can increase steady-state levels of PCS mRNA. In certain plants and fungi, increases in PCS expression upon metal exposure seems to be organism specific [2], but metal exposure is necessary to induce PCS expression and to produce phytochelatins for detoxification [1], [39]. Although we found an increase in PCS transcripts after exposure to metals, we could not detect any accumulation of phytochelatins down to the nM level. Because SmPCS enzyme activity is not influenced by cadmium (or other metals), it is likely that PCS functions constitutively and does enhance phytochelatin synthesis in the presence of metals. Moreover, the phytochelatin synthesis activity of the schistosome enzyme is low compared to plant PCS proteins (Fig. 2). Although it is clear from this and previous studies [11] that SmPCS can synthesize phytochelatins, it does not appear that in the parasite phytochelatins are synthesized for metal detoxification. Therefore, if phytochelatins are produced *in vivo*, they may be produced at low levels and may be involved in metal homeostasis rather than detoxification. In some organisms the cadmium detoxification system appears to be the result of a balance between antioxidant systems (antioxidant enzymes and GSH) and metal-specific detoxification systems (phytochelatins, metallothioneins) [40]. A number of studies have shown that metal stress is not necessarily counteracted by phytochelatin production [4]. Indeed, It is well known that although cadmium in not a redox metal, it can generate oxidative stress [13]. Iron is known to be a redox active metal [41]. Hence, it is likely the increase in mRNA level of PCS may be due to the oxidative stress triggered by cadmium and iron.

Because SmPCS can act as a peptidase on GSH conjugates, we followed transcriptional regulation of SmPCS in worms treated with compounds known to be eliminated through GST conjugation. We found that PCS mRNA levels increase in response to exposure suggesting that PCS is required to process those compounds. However praziquantel, which interacts with GST but is not processed by GST [42], [43], was also found to increase PCS level. Therefore, PCS seems to be up-regulated by an array of compounds and not only by those potentially directly involved in its activity. Studies have demonstrated that large gene sets are induced in response to various stressors/toxicants [44]. In general these studies have been used to identify particular genes involved in the detoxification process. In particular, cadmium exposure induces MAPK pathways that affect the expression of genes that detoxify related stressors notably in plants [45] and in invertebrates [46]. MAPK activation induces *c-jun* and *c-fos* and triggers, among others, genes related to metal trafficking and antioxidant defense. If those stressors activate MAPK pathway, and PCS is affected in response to this activation, it is likely that genes other than PCS are also affected.

GSH is involved in metal, antioxidant, and xenobiotic defenses through direct metal-thiol interactions, as a cofactor to transfer reducing equivalents to glutathione peroxidases, and through xenobiotic conjugation by GST [47]. We thus thought that PCS could be regulated as part of a comprehensive antioxidant GSH-dependent defense mechanism [22]. Metals have been shown to affect the transcript levels of γ-GCS in *Saccharomyces cerevisiae* [48] and in plants [49] and of PCS [50]. H_2_O_2_ was also found to trigger γ-GCS transcription in *S. cerevisiae* [48]. To verify this hypothesis, we looked at transcripts of proteins involved in GSH metabolism to see if PCS would follow the same trend of regulation. Our data show that various stressors (metal, xenobiotic, oxidative stress) enhanced γ-GCS transcription, supporting an increase of GSH metabolism under stress in the parasite, as it has been described in plants and yeast [51]. In schistosomes, GSH may be responsible for the direct detoxification of metal, which may explain why at high concentrations of metals PCS is inhibited *in vitro*. Since PCS uses GSH to synthesize phytochelatins, the cell spares GSH so that it can be use for direct metal detoxification and for counteracting the oxidative stress triggered by metals.

Other genes were found to be up-regulated with the stressors tested: γ-GT, as well as GSTs and TGR. Mammalian GSTs have been described to be up-regulated by products of oxidative stress [38]. Aragon *et al.* showed that praziquantel increases GST transcript abundance as well as other enzymes involved in antioxidant defense [32]. They suggested that the ‘anti-oxidant’ response may actually be induced by praziquantel. Interestingly, we found that praziquantel enhanced the transcription of the genes involved in GSH metabolism. All those data taken together tend to indicate that PCS may be up-regulated as part of the anti-oxidant response of the worms.

Using WISH, we localized PCS transcripts to the gut epithelium and esophageal gland in both adult male and female worms. This localization pattern suggests that PCS functions similarly in male and female schistosomes. It is well known that many proteases localize in the gut of *S. mansoni* where they participate in hemoglobin proteolysis [52] and cathepsin-L has been used as a gut epithelium marker [53]. The esophageal gland is responsible, among other activities, for secretions promoting the lysis of red cells after their ingestion by the parasite [54]. We hypothesize that because PCS mRNA, and possibly protein, localize to the esophageal gland and digestive tract that it is likely that PCS protein functions in the defense response triggered by compounds ingested by adult worms. In contrast, tissue-specific PCS expression in the nematode *Caenorhabditis elegans* is essentially nonoverlapping with that found *S. mansoni*. In *C. elegans*, expression was localized to the hypodermis, the pharyngeal grinder, the pharyngeal-intestinal valve, the bodywall and vulval muscles, and coelomocytes [55]. Different patterns of tissue expression suggest different functions for PCS proteins in nematodes and trematodes.

To conclude, we suggest that SmPCS synthesizes phytochelatins *in vivo* likely for metal homeostasis rather than detoxification. This is consistent with the fact that, whereas plants or earthworms live in soil contaminated with metals need such a system, *S. mansoni* does not face high concentration of metal during its residence in the human blood stream. Our data suggest that SmPCS may work in tandem with GSTs and γGT for xenobiotic degradation. SmPCS may be necessary to detoxify xenobiotics and/or oxidative stress that may be present in human blood. It is not clear if depriving the parasite of such a defense or control system may be deleterious for the parasite. Due to this bi-functionality, the role of SmPCS in GSH metabolism is however of great interest. Because PCS is absent from the human genome, understanding its function in the parasite may help to elucidate how the parasite deals with external stress and to target suitable enzymes or pathways.
